# The Extracellular Vesicles from the Commensal Staphylococcus Epidermidis ATCC12228 Strain Regulate Skin Inflammation in the Imiquimod-Induced Psoriasis Murine Model

**DOI:** 10.3390/ijms222313029

**Published:** 2021-12-02

**Authors:** Fernando Gómez-Chávez, Carlos Cedillo-Peláez, Luis A. Zapi-Colín, Guadalupe Gutiérrez-González, Isaí Martínez-Torres, Humberto Peralta, Leslie Chavez-Galan, Erick D. Avila-Calderón, Araceli Contreras-Rodríguez, Yaneth Bartolo-Aguilar, Sandra Rodríguez-Martínez, Mario E. Cancino-Diaz, Juan C. Cancino-Diaz

**Affiliations:** 1 Laboratorio de Enfermedades Osteoarticulares e Inmunológicas, Sección de Estudios de Posgrado e Investigación, Escuela Nacional de Medicina y Homeopatía, Instituto Politécnico Nacional, Mexico City 07320, Mexico; fergocha@gmail.com; 2 Laboratorio de Inmunología Experimental, Instituto Nacional de Pediatría, Secretaría de Salud, Mexico City 04530, Mexico; mvzcarloscedillo@hotmail.com; 3 Laboratorio de Inmunidad Innata, Departamento de Inmunología, Escuela Nacional de Ciencias Biológicas-Instituto Politécnico Nacional, Mexico City 07340, Mexico; ouch_lazc@hotmail.com (L.A.Z.-C.); jeulis_23@yahoo.com.mx (G.G.-G.); sandrarodm@yahoo.com.mx (S.R.-M.); 4 Departamento de Inmunología, Unidad de Investigación, Instituto de Oftalmología Conde de Valenciana, Mexico City 06800, Mexico; isaimtztorres@gmail.com; 5 Programa de Genómica Funcional de Procariotes, Centro de Ciencias Genómicas, Universidad Nacional Autónoma de México, Cuernavaca 04510, Mexico; peralta@ccg.unam.mx; 6 Laboratory of Integrative Immunology, Instituto Nacional de Enfermedades Respiratorias Ismael Cosió Villegas, Mexico City 14080, Mexico; lchavezgalan@gmail.com; 7 Laboratorio de Inmunomicrobiología, Departamento Microbiología, Escuela Nacional de Ciencias Biológicas-Instituto Politécnico Nacional, Mexico City 11340, Mexico; mosi6286@hotmail.com (E.D.A.-C.); aracelicontreras21@gmail.com (A.C.-R.); 8 Unidad Profesional Interdisciplinaria de Biotecnología del Instituto Politécnico Nacional (UPIBI-IPN), Mexico City 07340, Mexico; ybartolo@cinvestav.mx

**Keywords:** *Staphylococcus epidermidis*, extracellular vesicles, psoriasis, skin, imiquimod, proteomics

## Abstract

Extracellular vesicles (EVs) are evaginations of the cytoplasmic membrane, containing nucleic acids, proteins, lipids, enzymes, and toxins. EVs participate in various bacterial physiological processes. *Staphylococcus epidermidis* interacts and communicates with the host skin. *S. epidermidis’* EVs may have an essential role in this communication mechanism, modulating the immunological environment. This work aimed to evaluate if *S. epidermidis’* EVs can modulate cytokine production by keratinocytes in vitro and in vivo using the imiquimod-induced psoriasis murine model. *S. epidermidis’* EVs were obtained from a commensal strain (ATC12228EVs) and a clinical isolated strain (983EVs). EVs from both origins induced IL-6 expression in HaCaT keratinocyte cultures; nevertheless, 983EVs promoted a higher expression of the pro-inflammatory cytokines VEGF-A, LL37, IL-8, and IL-17F than ATCC12228EVs. Moreover, in vivo imiquimod-induced psoriatic skin treated with ATCC12228EVs reduced the characteristic psoriatic skin features, such as acanthosis and cellular infiltrate, as well as VEGF-A, IL-6, KC, IL-23, IL-17F, IL-36γ, and IL-36R expression in a more efficient manner than 983EVs; however, in contrast, Foxp3 expression did not significantly change, and IL-36 receptor antagonist (IL-36Ra) was found to be increased. Our findings showed a distinctive immunological profile induction that is dependent on the clinical or commensal EV origin in a mice model of skin-like psoriasis. Characteristically, proteomics analysis showed differences in the EVs protein content, dependent on origin of the isolated EVs. Specifically, in ATCC12228EVs, we found the proteins glutamate dehydrogenase, ornithine carbamoyltransferase, arginine deiminase, carbamate kinase, catalase, superoxide dismutase, phenol-soluble β1/β2 modulin, and polyglycerol phosphate α-glucosyltransferase, which could be involved in the reduction of lesions in the murine imiquimod-induced psoriasis skin. Our results show that the commensal ATCC12228EVs have a greater protective/attenuating effect on the murine imiquimod-induced psoriasis by inducing IL-36Ra expression in comparison with EVs from a clinical isolate of *S. epidermidis*.

## 1. Introduction

The delivery mechanisms of bacterial molecules to the host cells are a subject of extensive interest. The bacterial secretion systems release several molecules related to infection, competition, and communication [1]. These classical bacterial secretion systems release specific compounds; meanwhile, the massive release of bacterial molecules depends on the formation of extracellular vesicles (EVs) [2]. The EVs are formed from the evagination of the cytoplasmic membrane in Gram-positive or from the outer membrane in Gram-negative bacteria that can contain metabolites, proteins, lipids, nucleic acids, phages, enzymes, and toxins [3,4,5,6]. EVs may have a crucial role in bacterial physiology and ecology since they are involved in exchanging different molecules involved in the bacterial–host interaction, promoting survival, infection, invasion, immune evasion, and immune modulation [6,7]. The environmental conditions can regulate EV formation and release from Gram-positive bacteria [8,9]. Gram-positive bacteria EVs have been reported to be involved in cellular defense [10], cell communication [11], DNA transfer [12], pathogenesis by delivering virulence factors [13], and the inactivation of antimicrobials by enzymatic degradation [14].

In particular, *S. aureus* can use EVs for self-protection, releasing EVs containing β-lactamase (BlaZ), a protein that confers penicillin resistance [4,15]. Furthermore, *S. aureus*’ EVs can induce neutrophil cytotoxicity, increasing its survival when evaluate in animal models [16]. The application of *S. aureus*’ EVs to mouse skin exacerbates atopic dermatitis [17]. In contrast, mice immunized with *S. aureus*’ EVs induce protection against subcutaneous and systemic *S. aureus* infection [16]. *S. aureus* EV components can induce apoptosis or cytotoxicity, such as protein A and α-toxin [18,19]. Moreover, the EVs produced in a mouse pneumonia model by *S. aureus* [18] were found to inhibit the development of *S. epidermidis* biofilm but not the *S. aureus* biofilm, indicating a selective action [20]. In humans, the presence of *S. aureus*’ EVs has been detected in the serum of patients with osteomyelitis infected with this bacterium, but their role was not explored [21].

In contrast to the pathogenic *S. aureus*, *S. epidermidis* is an active inhabitant of the skin microbiome [22] with critical roles as a regulator of the skin immune response [23], as it can inhibit the establishment of pathogenic bacteria in the skin [24]. On the injured skin of subjects with psoriasis, *S. epidermidis* is under-represented, indicating that its absence may have a role in the development of psoriasis [25]; however, studies on the role of *S. epidermidis* in psoriasis are scarce. Psoriasis is the most common chronic inflammatory skin disease, characterized by epidermal hyperplasia (acanthosis) due to hyperproliferation and impaired differentiation of keratinocytes, scaling, and erythematous plaque formation, eventually resulting in loss of the protective skin barrier [26,27]. Psoriasis pathogeny depends on the inflammatory environment produced by the innate/adaptative cells and the skin inflammatory resident cells. In psoriasis, activated resident immune cells and keratinocytes can produce cytokines that initiate the inflammatory process such as IL-36, IL-23, and IL-22. Later, the environment induces the production of IL-17, TNF-α, IL-6, IL-8, and VEGF-A. These cytokines can exacerbate inflammation by the recruiting of T cells and neutrophils into dermis and epidermis [28,29].

Reports about the effects of *S. epidermidis’* EVs are scarce. In *S. epidermidis*-infected rats, as a model of osteomyelitis development, the presence of EVs was detected in their serum, but the way in which EVs influence the evolution of the disease was not evaluated [21]. As *S. epidermidis* participates in the regulation of the immune response of healthy skin, we hypothesized that *S. epidermidis’* EVs may regulate the immunological environment of psoriasis. Therefore, in this work, the immunoregulatory effect of EVs from commensal and clinical *S. epidermidis* strains on HaCat keratinocytes and psoriatic lesions using the imiquimod-induced psoriasis murine model was evaluated.

## 2. Results

### 2.1. Characterization of S. epidermidis EVs

**Figure 1:**
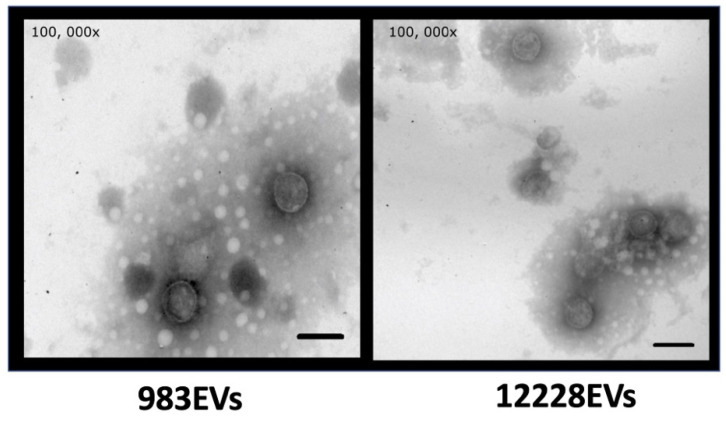
Extracellular vesicles (EVs) microphotographs from 983 and ATCC12228 *Staphylococcus epidermidis* strains. The bar corresponds to 50 μm.

EVs isolated from the *S. epidermidis* commensal and clinical strains are shown in Figure 1. The EVs from the 983 clinical strain (983EVs) sized on average 56.27 nm (27.7 to 135 nm), while EVs from *S. epidermidis* ATCC12228 (ATCC12228EVs) sized on average 66.9 nm (from 27.9 to 135 nm) (Figure 1). In both strains, EVs showed a spherical shape and a bilayer-lipidic membrane. The amount of protein was similar in both strains, 1.421 mg/mL for ATCC12228EVs and 1.377 mg/mL for 983EVs.

### 2.2. Effect of EVs on the Cytokine’s Expression in Human HaCaT Keratinocytes

**Figure 2:**
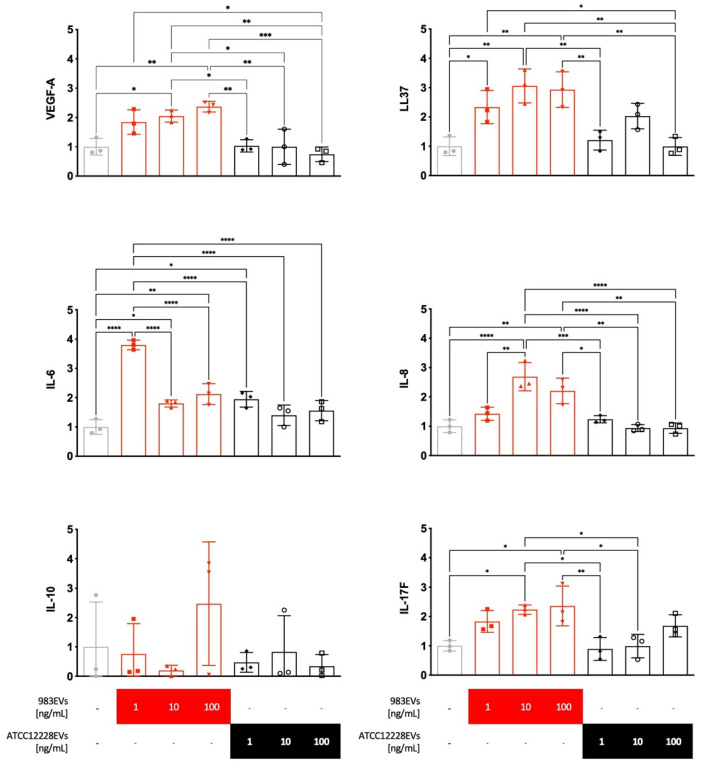
HaCaT keratinocytes stimulated with *Staphylococcus epidermidis* EVs. HaCaT keratinocytes were stimulated with EVs at different concentrations for 6 h. Subsequently, RT-qPCR was performed to measure the mRNA cytokine expression. Results show mean ± SD. ANOVA and Tukey’s post hoc analysis were performed for the expression of VEGF-A (F (6, 14) = 10.67; *p* = 0.0002), LL37 (F (6, 14) = 10.83; *p* = 0.0001), IL-17F (F (6, 14) = 7.135; *p* = 0.0012), IL-6 (F (6, 14) = 30.99; *p* < 0.0001), IL-8 (F (6, 14) = 17.36; *p* < 0.0001), and IL-10 (F (6, 14) = 1.262; *p* = 0.3349). The data were compared with the non-EV-stimulated control cells. * *p* ≤ 0.05, ** *p* ≤ 0.01, *** *p* ≤ 0.001, and **** *p* ≤0.0001.

The 983EVs, isolated from a clinical strain, significantly increased VEGF-A, LL37, IL-17F, and IL-6 mRNA expression at the different EVs concentrations evaluated compared to the non-stimulated HaCat keratinocytes (Figure 2). IL-8 mRNA expression was significantly induced only from 983EVs 10 and 100 ng/mL (Figure 2). Interestingly, the ATCC12228EVs did not induce significant changes in the expression of the cytokines mentioned above at any EVs concentrations tested (Figure 2), except for IL-6, which was increased at one ng/mL EVs concentration. The expression of IL-10 mRNA was not modified at any EV origin or concentration (Figure 2).

### 2.3. Effect of EVs on the Murine Imiquimod (IMQ)-Induced Psoriasis Model

**Figure 3:**
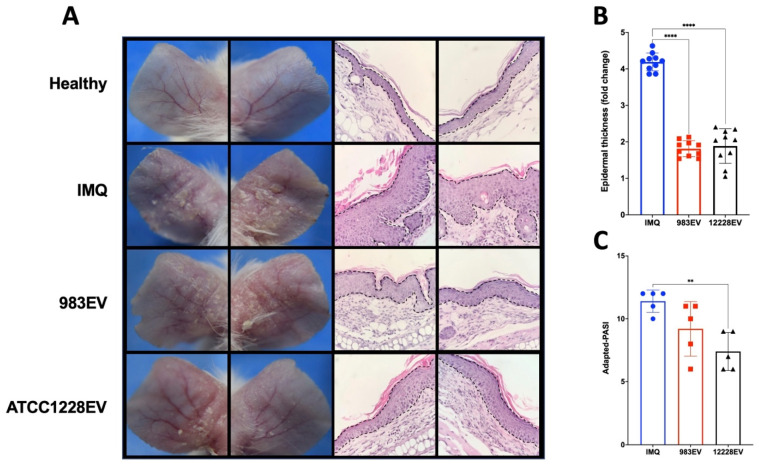
Imiquimod-induced psoriasis mice skin-treated with *Staphylococcus epidermidis* EVs. The ears of mice were treated topically with EVs (10 ng) 30 min before psoriasis was induced in the ears. Then, psoriasis was induced by imiquimod (IMQ); this procedure was performed for 6 days. Representative images of the ears and its histology (magnification, ×40) are shown (**A**), along with epidermal thickness ((**B**) F (2, 27) = 165.1; *p* < 0.0001) and adapted-PASI ((**C**) F (2, 12) = 7.718; *p* = 0.0070) at the end of treatment. Data are presented as mean ± SD, *n* = 10. ANOVA and Tukey’s post hoc analysis were performed. ** *p* ≤ 0.01, and **** *p* ≤ 0.0001.

In this murine psoriasis model, the topical IMQ treatment in the skin of the ears induce redness, skin thickness, and scaling with similar signs as occurring in the human psoriasis phenotype. Figure 3 shows the effect of EVs treatment in the ears of IMQ-induced psoriasis mice. EVs from both origins decreased the characteristic psoriatic phenotype observed in mice treated only with IMQ, with reduced cellular infiltrate and epidermal thickness (Figure 3A,B). However, the treatment with ATCC12228EVs showed a higher amelioration, reducing the degree of redness, epidermal thickness, and scaling than in the skin-like-psoriasis treated with 983EVs, as it is shown in the adapted-PASI score (Figure 3C).

### 2.4. ATCC12228EVs Decreased Cell Infiltration and Inflammatory Cytokine Expression in the IMQ-Induced Psoriasis Model

**Figure 4:**
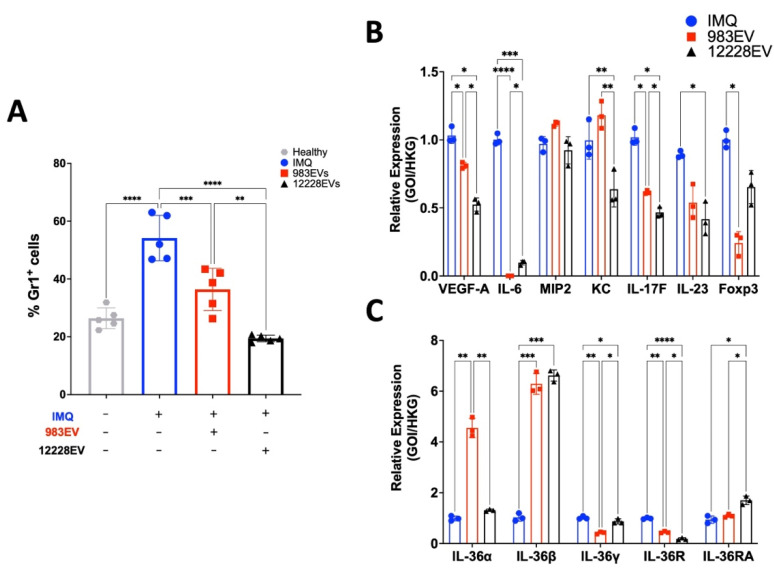
Neutrophil infiltrate and pro-inflammatory cytokine expression in the ears skin of EV-treated, IMQ-induced psoriasis mice. The mice ears from different treatments were processed to determine neutrophil infiltrate of Gr1^+^ cells ((**A**) F (3, 16) = 35.18; *p* < 0.0001) and pro-inflammatory cytokine expression levels ((**B,C**) F (6, 16) = 213.6; *p* < 0.0001). Data are presented as mean ± SD, *n* = 5. ANOVA and Tukey’s post hoc analysis were performed in comparison with the IMQ-treated mice; * *p* ≤ 0.05, ** *p* ≤ 0.01, *** *p* ≤ 0.001, and **** *p* ≤ 0.0001.

The recruitment of neutrophils as Gr1^+^ cells was evaluated in the ears of mice as an indication of the inflammation progress, as typically seen in psoriatic lesions. The IMQ-induced psoriasis ears of mice treated with ATCC12228EVs showed a significant decrease in the percentage of neutrophils, such as that found in healthy skin, an observation not found in those mice treated with 983EVs (Figure 4A).

On the other hand, the ATCC12228EVs treatment decreased the mRNAs skin expression of VEGF-A, IL-6, KC, IL-17F, IL-23 (Figure 4B), and the IL-36 family members IL-36γ and IL-36R (Figure 4C), compared to the IMQ-induced psoriasis skin mice with no EV treatment. In addition, IL-36α mRNA was expressed as in the IMQ control group, and IL-36β was induced (Figure 4B). In contrast, 983EVs treatment only significantly decreased IL-6, IL-17F, IL-36γ, and IL-36R, while the cytokines IL-36α and IL-36β were overexpressed compared to the only IMQ-induced psoriasis skin (Figure 4B). Furthermore, ATCC12228EVs did not significantly reduce the Foxp3 regulator expression but increased the IL-36 antagonist, the IL-36Ra, compared to the IMQ control group. In opposition, the 983EVs significantly inhibited Foxp3. In general, ATCC12228EVs reduced the IL-23, VEGF-A, KC, IL-17F, IL-6, and non-significantly stimulated IL-36-α, -γ, and -R expression compared to 983EV-treated mice.

### 2.5. Protein Identification in EVs

**Figure 5:**
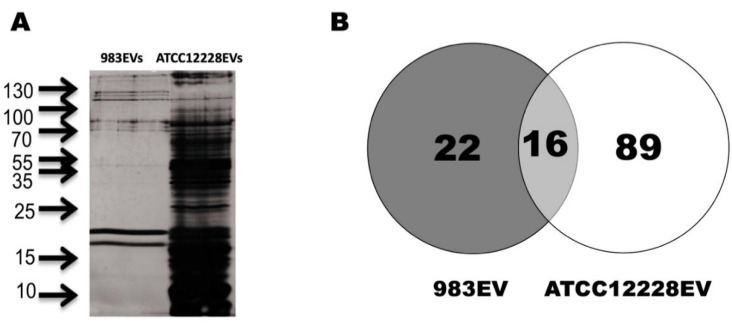
Protein profile and distribution of *Staphylococcus epidermidis* EVs. The EV total protein was separated on a 12% SDS-PAGE gel; arrows indicate the molecular weight marker (**A**). Venn diagram shows the distribution of the identified proteins by proteomics analysis in both EVs (**B**).

The electrophoretic protein profile of EVs obtained from 983 strain showed proteins from 10 to 130 kDa. On the other hand, three major band groups were observed in ATCC12228EVs: 1 (18–23 kDa), 2 (68–80 kDa), and 3 (115–130 kDa). A differential protein pattern between the ATCC12228EVs and the 983EVs is shown in Figure 5A. After electrophoretic separation, we performed a proteomic analysis to identify the protein composition of *S. epidermidis’* EVs.

The LC–MS/MS analysis identified 105 proteins in the 983EVs and ATCC12228EVs. From these proteins, 22 were exclusive for 983EVs, 89 for the ATCC12228EVs, and 16 proteins were shared between them (Figure 5B).

**Figure 6:**
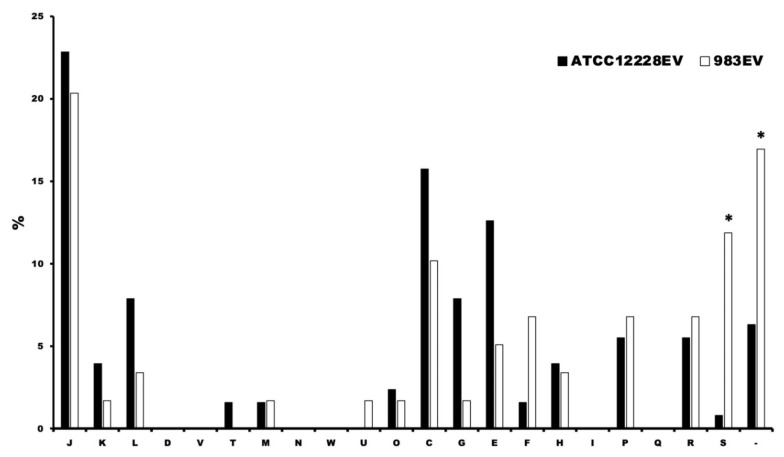
Functional comparison between *Staphylococcus epidermidis* EVs. COG J translation, COG K transcription; COG L replication and repair; COG D cell cycle control and mitosis; COG V defense; COG T signal transduction; COG M cell wall/membrane/envelop biogenesis; COG N cell motility; COG W extracellular structures; COG U intracellular trafficking and secretion; COG O post-translational modification, protein turnover, chaperone functions; COG C energy production and conversion; COG G carbohydrate metabolism and transport; COG E amino acid metabolism and transport; COG F nucleotide metabolism and transport; COG H coenzyme metabolism; COG I lipid metabolism; COG P inorganic ion transport and metabolism; COG Q secondary structure; COG R general functional prediction only; COG S function unknown. * Proportion analysis by the accurate Fisher test (*p* = 0.0015 for COG S, *p* = 0.0318 for not COG).

The identified proteins were assigned to their function key using the EggNOG program (Table 1 and Table 2). When the Clusters of Orthologous Groups of proteins (COG) functions of the proteins were compared between both EVs origins, we found that the 983EVs had more abundant proteins with unknown function (COG S) than in the ATCC12228EVs (Figure 6). Proteins with different functions were more abundant in the ATCC1228EVs, such as translation (COG J), transcription (COG K), replication and recombination (COG L), energy obtainment (COG C), carbohydrate metabolism (COG G), amino acid biosynthesis (COG E), coenzyme biosynthesis (COG H), inorganic ion transport/metabolism (COG P), and general activities (COG R). In the 983EVs, the proteins found were related to nucleotide synthesis (COG F) and intracellular trafficking and secretion (COG U). Importantly, we did not find any of the following functional classes in any of the EVs analyzed: cell division (COG D), defense mechanisms (COG V), secondary structure (COG Q), cell motility (COG N), extracellular structures (COG W), and lipid transport/metabolism (COG I).

### 2.6. Detection of Functional Protein Enrichment Using Gene Ontology Software

According to the Gene Ontology software, the identified proteins were subjected to an analysis to determine the functional enrichment of EVs. Analysis showed that 13 biological processes are shared between both vesicles (Table 3). The ATCC12228EVs had proteins with exclusive biological processes, including metabolic process, catabolic process, tricarboxylic acid cycle, acetyl-CoA catabolic process, macromolecule biosynthetic process, and coenzyme catabolic process. The 983EVs had exclusive biological processes such as pathogenesis, cellular process, gene expression, multi-organism process, primary metabolic process, and nucleobase metabolic process (Table 3).

For molecular function (Table S1), we found that the proteins of both vesicles have functions like a structural constituent of ribosome, structural molecule activity, rRNA binding, and RNA binding. ATCC12228EVs have proteins involved in tRNA binding, oxidoreductase activity, activity on the amino acid, nucleic acid binding, oxidoreductase activity, and activity on CH–OH. Other molecular functions were found to not have significant representation.

From all these data and the analysis of the metabolic processes, we can conclude that the proteins in each vesicle could participate in essential functions of the cell, with ATCC12228EVs having a higher number of proteins. Some proteins present in ATCC12228EVs could suggest an interesting role such as HTH-type transcriptional regulator rot, universal stress protein UspA, penicillin-binding protein 2, glycosyltransferase, 60 kDa chaperonin, ornithine carbamoyltransferase, arginine deiminase, glutamate dehydrogenase, carbamate kinase, catalase, superoxide dismutase, phenol soluble modulin β1/β2, and poly (glycerol-phosphate) α-glucosyltransferase. In the case of 983EVs, the proteins found were antiholin-like protein LrgA, N-acetylmuramoyl-L-alanine amidase, antibacterial protein 2, and siphovirus Gp157.

## 3. Discussion

Bacterial EV research has concentrated on Gram-negative bacteria, and less is known about Gram-positive bacteria EV production and function. Concerning *S. epidermidis*, progress on EV research is scarce. It is well known that *S. epidermidis* contributes to human skin homeostasis [30], and its interaction with the skin is evolutionarily beneficial for both organisms [31]. The mechanism of communication between this bacterium and the skin is not yet known in detail. This work suggests that communication between *S. epidermidis* and the host skin may also occur by releasing bacterial products transported in EVs.

In this work, we obtained spherical-double membrane *S. epidermidis’* EVs, with a diameter around 66.9 nm, similar to those reported in vitro and in vivo by Zaborowska et al. (2020) and Deng et al. (2020) [21,32]. It was also demonstrated that EVs from a commensal isolate (ATCC12228) and a clinical isolate (983) have the same morphology but a different protein content, even if they are obtained under the same culture conditions, and they can differentially modulate cytokine production on human keratinocytes and mice psoriatic-like skin.

The ATCC12228EVs significantly reduced the expression of LL37, IL-6, IL-23, IL-17, KC, IL- 36R, and IL-36Ra. All these molecules are essential in the initiation and development of the mice imiquimod-induced psoriasis and human psoriasis. In the context of skin inflammation, LL37 is overexpressed in the skin of psoriatic patients compared to healthy skin [33]. The LL37 overproduction is associated with the onset of psoriasis by the damaged keratinocytes releasing LL37 and genomic DNA or RNA. Then, DNA-LL37/RNA-LL37 complexes are recognized by plasmacytoid dendritic cells (pDC) and myeloid DC (mDC) inducing type I IFN expression, as well as TNFα, IL-6, and IL-36γ [34,35,36]. Furthermore, LL37 induces the chemokines CXCL8 and CXCL1 through the IL-36R signaling in psoriatic keratinocytes, leading to neutrophils’ recruitment [36]. Other cytokines such as IL-6 and IL-23 can promote a Th17 environment, favoring skin inflammation along the IL-23/IL-17 axis [37]. Furthermore, IL-6 inhibits the TGF-β-dependent differentiation of Treg cells [38] and induces mononuclear cells/macrophages IL-8 and MCP-1 production [39], resulting in more neutrophils recruiting. On the other hand, IL-6 is also capable of enhancing keratinocyte growth and proliferation [40]. A wide variety of cell types can produce IL-6 in response to stimulation by IL-36 [40]. IL-6 and IL-23 can stimulate IL-17F production in lesioned psoriatic skin compared with non-lesioned skin in humans, and it has been reported as one of the central cytokines in psoriasis development [41]. The axis formed by IL-23/IL-17/IL-22 was thought to be the trigger of psoriasis. However, the loss of IL-36R signaling successfully counteracts and protects against the development of imiquimod-induced psoriasis compared to deletions of the IL-23/IL-17/IL-22 axis, indicating that IL-36 activity should be early in the cascade of psoriasis initiation [42,43,44,45]. Importantly, IL-36R signaling in keratinocytes is crucial for the early production of IL-23, IL-17, and IL 22 and neutrophil infiltration [44,45]. The IL36 family members, the IL-36α, β, and γ, are highly expressed in psoriatic lesions and influence the function of DCs [46]. In humans, IL-36 has been shown to activate mDCs, stimulating the secretion of IL-1β and IL-6, promoting the differentiation of the Th17 cells [47]. Imiquimod via TLR-7 induces the expression of IL-36α in the keratinocyte, and this stimulation drives IL-23 and Th17-related cytokine/chemokine production [48]. TNF-α induces IL-36γ in psoriatic lesions, which promotes the expression of antimicrobial peptides and chemokines and Th17 cells and interferes with terminal differentiation and the cornification process of the psoriatic epidermis [49]. Furthermore, pretreatment with IL-36Ra has a protective effect on imiquimod-induced psoriasis and saves mice from the severe disease phenotype [50]; in addition, mice deficient in IL-36Ra show exacerbated psoriasiform disease [42]. Finally, VEGF-A is an angiogenic cytokine increased in psoriatic tissues that promote the hyperproliferation of keratinocytes [51]. Accordingly, keratin 14-VEGF-A transgenic mice develop psoriasis [51,52], indicating its critical role in the disease.

ATCC12228EVs decreased the expression of IL-36R and IL-36α but increased the IL-36Ra, suggesting that the mechanism of action for reducing imiquimod-induced psoriasis depends on the downregulation of the IL-36R signaling, in general attenuating the development of the psoriatic lesion. On the other hand, 983EVs showed a lower effect over the cytokine expression, and its most evident effect was found in the reduction of the master regulator of Treg cells (Foxp3), but it was not as effective in reducing the development of psoriatic lesion as it did the ATCC12228EVs. What is evident is that *S. epidermidis* vesicles have an anti-inflammatory effect on the skin compared to the cutaneous inflammatory effect of *S. aureus* vesicles reported elsewhere [17]. These results suggest that EVs from different origins or strains (commensal or clinical), grown under the same conditions, can differentially regulate cytokine production in the skin.

To our knowledge, this is the first report about proteomics in *S. epidermidis’* EVs. Surprisingly, EVs composition analysis revealed that some proteins detected in *S. epidermidis’* EVs have been reported in *S. aureus*’ EVs, highlighting the idea that some proteins are shared in the EVs of these two staphylococcal species. In 983EVs, we detected proteins such as the N-acetylmuramoyl-L-alanine amidase protein [4,53], ATP synthase subunit β [4,53], pyruvate dehydrogenase E1 component subunit α [4,18], dihydrolipoyllysine-residue acetyltransferase component of pyruvate dehydrogenase complex, 30S ribosomal protein S3, and 30S ribosomal protein S4 [4]. In ATCCC12228EVs, we found proteins such as the penicillin-binding protein 2 [4,18,53], ATP synthase subunit β [4,53], glutamine synthetase [53], pyruvate kinase, DNA-directed RNA polymerase subunit β [53], 30S ribosomal protein S7, 50S ribosomal protein L5, 50S ribosomal protein L1 [4], catalase (560), enolase [53], phenol-soluble modulin β 1, superoxide dismutase [53], HTH-type transcriptional regulator rot [16], glycosyltransferase, 60 kDa chaperonin, and glutamate dehydrogenase [53]. Previously, Silva Ribeiro et al. reported that dramatic differences in protein EVs from two distinct *Trypanosoma cruzi* strains may correlate with their infectivity/virulence during the host–parasite interaction. Thus, differences between specific proteins in 983EVs and ATCC12228EVs could determine the behavior as a clinical isolate and a commensal strain [54].

Regarding the regulation of psoriasis by *S. epidermidis* EVs, it is difficult to precisely denote which proteins are functioning as regulators of the inflammation in the psoriasis animal model since the proteome of the vesicles mainly identified proteins with participation in the essential metabolism, which was expected since *S. epidermidis* does not have many virulence factors as is the case for *S. aureus* [55].

We hypothesized that some proteins present in the commensal strain ATCC12228EVs that are not contained in 983EVs could regulate inflammation, avoiding the development of psoriatic lesions. However, there are no precedents that support this suggestion. Nevertheless, the presence of enzymes involved in the glutamate and glutamine metabolism are present in ATCC12228EVs, and these could be relevant participants that interfere in the production of characteristic plaques in this disease. It is known that amino acids, essential constituents of the skin’s natural moisturizing factors, are decreased in psoriasis [56]. A metabolic study of psoriasis patients showed that their serum has high α-ketoglutarate levels, glucuronic acid, and a low level of asparagine and glutamine [57]. Glutamate dehydrogenase could convert α-ketoglutarate to glutamate, and glutamate is a source to produce glutamine by the enzyme glutamine synthetase, with both enzymes being present in ATCC12228EVs. The enzyme glutamate-1-semialdehyde 2,1-aminomutase, also present in the ATCC12228EVs, converts L-glutamate 1-semialdehyde to the d-aminolevulinic acid, which is used for the treatment of psoriasis by photodynamic therapy with favorable results in humans [58]. Furthermore, *S. epidermidis* can convert aromatic amino acids into trace amines (TA), which accelerate skin wound healing [59].

The enzymes catalase and superoxide dismutase (presents ATCC12228EVs) may participate in the attenuation of reactive oxygen species (ROS) compounds. Liu et al. reported decreased levels of reduced glutathione (GSH), superoxide dismutase (SOD), and catalase (CAT), as well as the accumulation of malondialdehyde (MDA), in the skin of IMQ-induced psoriasis [60]. In this way, catalase and SOD from ATCC12228EVs could contribute to the reduction of ROS in psoriasis.

Other proteins present in ATCC12228EVs could ameliorate IMQ-induced psoriasis; for example, glycine dehydrogenase converts glyoxylate to glycine, and glycine has antioxidative and anti-inflammatory effects regulating apoptosis in various models [61]. The soluble phenol modulin (PSM) β1/β2 are related to biofilm maturation and removal [62], and it is also reported that these PSM regulate the release of vesicles in *S. aureus* [53]. Poly (glycerol-phosphate) α-glucosyltransferase participates in the synthesis of lipoteichoic acid (LTA). LTA can be released into the environment, modulating the keratinocyte immune response [63] and regulating the pathogenesis of *Cutibacterium acnes* [24].

The use of beneficial bacteria as probiotics for the skin represents another alternative method for treating skin diseases, such as activated *Lactobacillus acidophilus* [64], *L. paracasei*, *L. brevis*, or *L. fermentum*, which are ingredients in skincare products [65], as well as *Streptococcus salivarius* K12, hich stimulates an anti-inflammatory response in the skin [66]. A bacterial extract of the Gram-negative bacteria *Vitreoscilla filiformis* showed a beneficial effect when applied to lesions of patients with atopic dermatitis [67]. A note of caution is due here since bacterial extracts (cytoplasmic proteins) in some cases can have adverse effects, and more research in this area is needed.

## 4. Materials and Methods

### 4.1. EV Isolation

*S. epidermidis* clinical strain 983 was isolated from a corneal infection; it is an ST2 genotype and biofilm producer [68]. EVs from 983 and ATCC12228 strains were obtained according to Avila-Calderón et al. (2012) and Ruiz-Palma et al. (2021) under the same culture conditions [69,70]. Briefly, both strains were cultured on supplemented LB agar plates and incubated for 24 h at 37 °C. A bacterial suspension was grown up and cultured in bulk in 25 LB agar plates from the pure plate colonies, and then these plates were incubated for another 24 h at 37 °C. Bacteria culture was harvested with a sterile cell scraper and suspended in 200 mL of sterile phosphate saline buffer (PBS). The bacterial suspension was centrifuged at 10,000× *g* for 30 min to remove intact cells. The supernatant was filtered through a 0.22 µm pore filter (Millipore Corp., Billerica, MA, USA) to remove the remaining bacteria. Then, the supernatant was cultured on LB agar plates for 48 h at 37 °C to evaluate sterility. Then, the sterile supernatant was ultracentrifuged at 100,000× *g* for 2 h at 4 °C. The pellet containing the EVs was washed twice with 25 mL of sterile PBS. In the last washing, the EVs were suspended in 1 mL of sterile PBS. The EVs were finally purified by density gradient using OptiPrep (Sigma-Aldrich, Inc., Burlington, MA, USA). OptiPrep was diluted to final concentrations of 10, 15, 20, 25, and 30% in sterile PBS. Later, 2.6 mL of each OptiPrep solution was layered from the higher to the lower density in an ultracentrifuge tube, and the EVs were loaded at the top of the tube. The samples were centrifuged at 100,000× *g* for 16 h at 4 °C. EVs appeared as an opalescent band in the density gradient. EVs were collected, washed twice with sterile PBS, and centrifuged at 100,000× *g* for 2 h at 4 °C, and finally suspended in 300 μL of sterile PBS. This procedure was performed in three independent experiments for each strain. The total protein concentration was determined using PIERCE-BCA (Thermo-Fisher Scientific Inc., Waltham, MA, USA). The EV samples were divided into 0.5 mL aliquots and stored at −80 °C until used.

### 4.2. Observation of EVs by Electron Microscopy

A total of 20 µL of purified EVs equivalent to 25 µg from both strains were placed onto copper grids coated with formvar and dried using filter paper. One percent of phosphotungstic acid was added, and the grids were allowed to dry for 10 h at room temperature. All preparations were stained and observed in a transmission electron microscope (JEOL model JEM 10–10).

### 4.3. Stimulation of HaCaT Keratinocytes with S. epidermidis EVs

We cultured 1 × 10^6^ HaCaT keratinocyte cells in 6-well plates until 80% confluence. Before the stimulus, keratinocytes were washed with sterile 0.1 M PBS (pH 7.4) and with 1X DMEM (Life Technologies, CA, USA). Subsequently, we added to the cultures 800 µL of DMEM/F12-K (1:1) supplemented with 10% FBS and 1X antifungal antibiotic (Life Technologies, CA, USA). Keratinocyte cultures were stimulated with *S. epidermidis* EVs at the final concentrations of 1, 10, and 100 ng/mL and incubated for 6 h at 20% O_2_, 5% CO_2_, and 37 °C. Three independent experiments were carried out, and unstimulated cells were used as the control group.

### 4.4. HaCaT Keratinocyte RNA Extraction, cDNA Synthesis, and PCR

Total RNA was obtained from stimulated and unstimulated keratinocytes using the TRIzol reagent (Invitrogen, CA, USA). RNA purification and RT-qPCR were performed as previously described [71]. Briefly, cells were washed with 1× PBS, and total RNA was extracted with TRIzol (Invitrogen), treated with DNase I (Invitrogen), and then re-extracted. For the reverse transcriptase (RT) reaction, 3 µg of total RNA was denatured at 70 °C for 10 min in the presence of 0.5 μg of oligo-hexamers (Invitrogen). Then, we added 1× single strand buffer, 0.5 mM DTT, 10 mM of each dNTPs, and 200 U of MMLV reverse transcriptase (Invitrogen). Reverse transcriptase reactions were performed at 42 °C for 1 h. The PCR conditions for LL37, VEGF-A, IL-6, IL-17F, and IL-8 were performed as follows: 40 cycles of denaturation at 94 °C 30 s, hybridization at 60 °C 30 s, elongation at 72 °C 30 s. Endogenous gene (GADPH): 28 cycles of denaturation at 94 °C 30 s, hybridization at 60 °C 30 s, and elongation at 72 °C 30 s. The oligonucleotide sequences used for amplification are shown in Table S2. Relative expression was determined by the 2^−ΔΔCt^ method. The results are expressed as means ± standard deviation of triplicate assays.

### 4.5. Imiquimod (IMQ)-Induced Murine Psoriasis Model Treated with Staphylococcus Epidermidis EVs

All the mice used in this work were supplied by the Animal Care Facility of the Escuela Superior de Medicina-IPN. We used female Balb/c mice (eight weeks old; 22–25 g), housed at 23 °C under 12 h light/dark cycles, 40–60% humidity, and ad libitum access to food/water. Two independent experiments were conducted with 20 mice, grouped as follows: control untreated healthy (*n* = 5), IMQ-treated (IMQ; *n* = 5), ATCC12228EV/IMQ-treated (*n* = 5), and 983EV/IMQ-treated (*n* = 5). To evaluate the EV effect on psoriasis-like skin, we depilated mice ears, and 30 min before the treatment with Aldara^TM^ (6.25 µg, IMQ 5%, Graceway Laboratory), the EVs (10 ng of total protein) were topically administered using the same vehicle as used in the Aldara^TM^ cream. This procedure was performed once a day for six days, in accordance with the work of Van der Fits et al. (2009) [72]. After the third day of IMQ administration in the only IMQ-treated group, the psoriasis-like lesions were visible and clearly defined after the sixth day of treatment. On the sixth day of treatment, the redness, scaling, and thickness of mice were evaluated to score the “adapted-PASI”, which assesses the severity of the induced murine psoriasis erythema, desquamation, and induration; these severity parameters are measured on a 0–4 scale (from none to the maximum damage). The sum of these four values is the value of the adapted-PASI. An adapted-PASI value of 12 indicates a maximum severe injury. On day 7, mice were euthanized with an overdose of pentobarbital sodium (150 mg/kg) by intravenous injection, and the ears were collected for histochemistry, flow cytometry, and RT-qPCR analysis. The handling of the ear samples was as follows: both ears of a mouse were divided into two equal parts. The one-half ear was used for histochemistry, and the other half for the determination of GR1 + cells by flow cytometry. The second ear of the same mouse was used for histochemistry and the other half ear for RT-qPCR analysis. Statistics were calculated using 10 data per group for histochemistry and 5 data per group for GR1 + cell determination and RT-qPCR.

### 4.6. Histochemistry

The ears sections used for histochemistry analysis were maintained in a fixative buffered formalin solution (10% in PBS) until used. The skin was placed into cassettes and dehydrated using an ethanol series (70, 80, 90, 96, 100, and 100% for 1 h each). Tissues were cleared twice in 100% xylene for 1 h and soaked in paraffin twice at 60 °C for 1 h. Subsequently, tissues were sectioned in 5 µm slices using a Leica RM 2132 microtome (Leica Microsystems GmbH). The slices were stained in hematoxylin and eosin (H&E) and Lillie’s trichrome solutions for 5 min at room temperature. Stained sections were observed in 10 randomly selected fields in a transmitted light microscope (magnification ×40). Finally, we performed a blind histological examination and interpretation, and photographs were taken to determine the size of the epidermal thickness using ImageJ [73].

### 4.7. Skin RT-PCR

The mice’s ears skin was cut and homogenized individually with a mortar and pestle in Trizol. Total RNA yield was around 10–30 μg per a half mice ear of ≈15–30 mg. Then, cDNA synthesis was performed following the same procedure mentioned above to detect GADPH, VEGF-A, IL-6, MIP2, KC, IL-17F, IL-23, Foxp3, IL-36α, IL-36β, IL-36γ, IL-36R, and IL-36 receptor antagonist (IL-36Ra) (Table S2). Relative expression was determined by the 2^−ΔΔCt^ method. The results were expressed as means ± standard deviation of triplicate assays.

### 4.8. GR1^+^ Cell Detection in the Skin of Ears from Treated Mice

Ear skin sections were placed on a plate with 1 mL of RPMI. The inner and outer faces of the ears were separated and incubated in RPMI supplemented with collagenase type I (60 U/mL; Life Technologies, NY, USA), liberase TL (153.5 μg/mL; Roche Diagnostics, Mannheim, Germany), and DNAse I (6.5 ng/mL; Roche Diagnostics) for 2 h at 37 °C. After incubation, both faces were fragmented, transferred to 1 mL of fresh medium supplemented with enzymes, and incubated for 30 min more at 37 °C. The tissues were homogenized and filtered at 70 µm; the cell suspension was rinsed with 1 mL of FACS buffer, centrifuged for 5 min at 450× *g*, and finally resuspended in 500 μL of FACS buffer. A total of 1 × 10^6^ cells were stained with 100 μL of anti-CD45 PE-Cy5 antibody (1:100) (monoclonal antibody rat anti-mouse; Biolegend, CA, USA) and anti-GR1 Alexa Flour 488 antibody (1:100) (monoclonal antibody rat anti-mouse; Biolegend), and then incubated on ice for 30 min. The cells were then washed with FACs buffer and resuspended in 200 μL of FACs buffer. All samples were acquired in a FACSAria II cell sorter (BD Bioscience; Figure S1).

### 4.9. Protein Identification by Liquid Chromatography Coupled with Tandem Mass Spectrometry (LC–MS/MS)

EVs were mixed with the SDS-PAGE Leammli loading buffer, boiled for 90 s, and centrifuged at 10,000× *g* for 5 min to eliminate insoluble fraction. A total of 100 µg of soluble protein fractions were loaded to 12% SDS-PAGE. Proteins were visualized by staining with Coomassie blue. Then, gels were destained in a 50:50 (*v*/*v*) solution of 100 mM, pH 7.8 ammonium bicarbonate (Sigma-Aldrich, Mexico Edo, Mexico) and acetonitrile treated with 50 mM dithiothreitol (DTT, Sigma-Aldrich), alkylated with 50 mM iodoacetamide (Sigma-Aldrich). An “in-gel” digestion was performed using 10 ng/μL trypsin (Promega Sequencing Grade Modified Trypsin, WI, USA). The peptides were desalted using ZipTip^®^ C18 (Merck KGaA, Darmstadt, Germany) solution and then concentrated in a Speed-Vac SPD 1010 Thermo-Electron. This procedure was performed in three independent experiments for each EVs.

The proteomic analysis was performed in the Proteomics Facility of the Instituto de Biotecnología-UNAM, Cuernavaca, Mexico. The peptide mixtures were eluted in 50% acetonitrile containing 1% acetic acid and loaded into a liquid chromatography system coupled to mass spectrometry (LC–MS/MS), consisting of an EASY-nLC II nanoflow pump, coupled with an LTQ-Orbitrap Velos (Thermo-Fisher; MA, USA) mass spectrometer with a nano-electrospray (ESI) ionization source. For nanoflow chromatography, a gradient system of 5–80% solvent B (water/acetonitrile with 0.1% formic acid) and solvent A (water with 0.1% formic acid) using a capillary column (ID 0.75 µm × 10 cm, RP-C18) with a flow rate of 300 nanoliters/min, in a time of 120 min, was used.

We performed an average mass scan of the total ions on the Orbitrap analyzer with a 60,000 (RP = m/FWHM) mass resolving power. The CID (collision-induced dissociation) and HCD (high-energy collision dissociation) methods were applied with a 15,000 (RP = m/FWHM) mass resolving power.

The protein analysis was performed using the Proteome Discoverer 1.4 program (Thermo Fisher) through the Sequest H and X! Tandem search engines. We configured the Sequest HT search engine to identify the *S. epidermidis* protein database (UniProt). The precursor ion’s mass tolerance and the ion fragments obtained by dissociation of the precursor ion were 20 ppm and 0.6 Da. MW using an FDR (false discovery rate) of 0-01 and 0.05 (maximum). We worked with the inverted database (Decoy database), the Scaffold program (version 4.8.9) as a tool of the “Percolator” validation program for the validation of the peptides, and protein identification based on MS/MS, establishing for the identification of the peptides a probability higher than 96% to achieve an FDR less than 5%, and for the identification of the proteins a probability higher than 93% to reach an FDR less than 1%. The Protein Prophet algorithm assigned the probabilities.

### 4.10. In Silico Analysis

The proteins identified by LC–MS/MS were related to their closest homolog of *S. epidermidis* strains RP62A and ATCC12228 by BLAST analysis (http://blast.ncbi.nlm.nih.gov, accessed on 8 March 2021) with its default parameter settings. The protein accession numbers were obtained in strains RP62A and ATCC12228 from the Uniprot database (http://www.uniprot.org/uniprot, accessed on 8 March 2021). The clusters of orthologous groups’ (COG) functional group were assigned using Eggnog (http://eggnog.embl.de, accessed on 8 March 2021), version_3.0. The participation in metabolic pathways were obtained using the Kyoto Encyclopedia of Genes and Genomes (KEGG; http://www.genome.jp/kegg, accessed on 8 March 2021).

### 4.11. Statistics

To determine the proportion significant differences, we used the accurate Fisher test. To analyze differences in the expression levels of the cytokines measured, we conducted a two-way ANOVA and a Tuckey post hoc test. These analyses were carried out with the software GraphPad Prism version 7.0 (GraphPad, San Diego, CA, USA).

## 5. Conclusions

Commensal *S. epidermidis’* EVs can modulate inflammation and psoriasis development in part by the axis IL-36R and IL-36Ra. We suggest that bacterial extracellular vesicles could be a cleaner and safer new alternative for dermatosis treatment.

## Figures and Tables

**Table 1:** Proteins found in ATCC12228EV.

Class	Accession	COG Number	Gene Name	Function
J	Q8CQV5	COG1190	lysS	Lysine–tRNA ligase
J	Q8CSD5	COG0423	glyQS	Anticodon-binding domain protein
J	Q8CSY9	COG0016	pheS	Phenylalanine–tRNA ligase alpha subunit
J	Q8CRH9	COG0200	rplO	50S ribosomal protein L5
J	Q8CS87	COG0261	rplU	50S ribosomal protein L21
J	Q8CRI3	COG0099	rpsM	30S ribosomal protein S13
J	P66618	COG0049	rpsG	30S ribosomal protein S7
J	Q8CRH2	COG0094	rplE	50S ribosomal protein L5
J	Q8CRH1	COG0198	rplX	50S ribosomal protein L24
J	Q8CTT4	COG0081	rplA	50S ribosomal protein L1
J	Q8CRH7	COG0098	rpsE	30S ribosomal protein S5
J	Q8CRG2	COG0089	rplW	50S ribosomal protein L23
J	Q8CS77	COG0292	rplT	50S ribosomal protein L20
J	Q8CRI5	COG0203	rplQ	50S ribosomal protein L17
J	Q8CTT5	COG0080	rplK	50S ribosomal protein L11
J	Q8CRJ0	COG0103	rpsI	30S ribosomal protein S9
J	Q8CRG4	COG0185	rpsS	30S ribosomal protein S19
J	P66336	COG0051	rpsJ	30S ribosomal protein S10
J	Q8CRH6	COG0256	rplR	50S ribosomal protein L18
J	Q8CRH4	COG0096	rpsH	30S ribosomal protein S8
K	Q8CQ84	COG0085	rpoB	DNA-directed RNA polymerase subunit beta
K	Q8CNU6	COG1846	Rot	HTH-type transcriptional regulator rot
K	Q8CRX8	COG1476	Cro	Cro/Cl family transcriptional regulator
K	Q8CRJ1	COG1349	lacR	Lactose phosphotransferase system repressor
L	Q8CNN0	COG0582	tnpA	Transposase A
L	Q8CP25	COG0648	Nfo	Probable endonuclease 4
L	Q8CPC6	COG0420	sbcD	Exonuclease SbcCD, C subunit
L	Q8CP04	COG0507	-	Helicase, RecD/TraA family
L	Q8CRP6	COG0513	cshA	DEAD/DEAH box helicase domain protein
L	Q8CNX7	COG0258	polA	DNA polymerase I
L	Q8CPT9	COG1074	addA	Helicase-exonuclease AddAB, AddB subunit
L	Q8CNX7	COG0258	polA	DNA polymerase
L	Q8CPZ0	COG0556	uvrB	UvrABC system protein B
T	Q8CS61	COG0589	uspA	Universal stress protein UspA
T	Q8CQK0	COG0745	walR	Transcriptional regulatory protein WalR
M	Q8CP74	COG0744	-	Penicillin-binding protein 2
M	Q8CMV0	COG0438	gtf1	Glycosyltransferase
O	P0C0N7	COG0459	groEL	60 kDa chaperonin
O	Q8CTA6	COG0396	-	ABC transporter (ATP-binding protein)
C	Q8CN04	COG1012	rocA	1-Pyrroline-5-carboxylate dehydrogenase
C	Q8CSL9	COG0508	odhB	Dihydrolipoyllysine-residue succinyltransferase
C	Q8CPH5	COG0045	sucC	Succinyl-CoA ligase (ADP-forming) subunit beta
C	Q8CNX4	COG0538	Icd	Isocitrate dehydrogenase (NADP)
C	Q8CP83	COG0567	odhA	2-Oxoglutarate dehydrogenase E1 component
C	Q8CPL2	COG1053	sdhA	Succinate dehydrogenase flavoprotein subunit
C	Q8CQA3	COG1249	lpdA	Dihydrolipoyl dehydrogenase
C	Q8CT13	COG0508	odp2	Dihydrolipoyllysine-residue acetyltransferase component of pyruvatedehydrogenase complex
C	Q8CNJ7	COG0055	atpD	ATP synthase subunit beta
C	Q8CS25	COG1866	pckA	Phosphoenolpyruvate carboxykinase (ATP)
C	Q8PCP2	COG1048	acnA	Aconitate hydratase1
C	Q8CNI5	COG1012	-	Aldehyde dehydrogenase (NAD) family protein
C	Q8CNJ1	COG0356	atpA	ATP synthase subunit alpha
C	Q8CN24	COG1012	aldA	Aldehyde dehydrogenase (NAD) family protein
C	Q8CQB2	COG0371	gldA	Glycerol dehydrogenase
C	Q8CQA1	COG0022	-	Branched-chain alpha-keto acid dehydrogenase E1
C	Q8CMZ0	COG0039	idh2	L-Lactate dehydrogenase
G	Q8CPY5	COG0057	Gap	Glyceraldehyde 3-phosphate dehydrogenase, C-terminal domain protein
G	Q8CPY3	COG0148	Eno	Enolase
G	Q8CTD6	COG0126	Pgk	Phosphoglycerate kinase
G	Q8CS69	COG0469	Pyk	Pyruvate kinase
G	Q8CN17	COG1869	rbsD	D-ribose pyranase
G	Q8CRJ4	COG1105	lacC	Tagatose-6-phosphate kinase
G	Q8CN27	COG3855	Fbp	Fructose-1,6-bisphosphatase class 3 O
G	Q8CPC7	COG0021	Tkt	Transketolase
E	P0C0N1	COG0078	argF	Ornithine carbamoyltransferase
E	Q8CU41	COG0078	arcB	Ornithine carbamoyltransferase
E	Q8CSR8	COG0174	glnA	Glutamine synthetase
E	Q8CQG5	COG2235	arcA	Arginine deiminase
E	Q8CPU5	COG0334	gdhA	Glutamate dehydrogenase
E	Q8CSR8	COG0174	glnA	Glutamine synthetase
E	Q8CU41	COG0078	otcC1	Ornithine carbamoyltransferase
E	Q8CMM1	COG1003	gcvPB	Glycine dehydrogenase subunit 2
E	Q8CMM0	COG0403	gcvPA	Probable glycine dehydrogenase (decarboxylating) subunit 1
E	Q8CP09	COG0169	aroE	AroE
E	Q8CPN0	COG3842	potA2	ABC transporter, ATP-binding protein
E	Q8CU42	COG0549	arcC2	Carbamate kinase
E	Q8CTA4	COG0520	Csd	Cysteine desulfurase
E	Q8CNQ9	COG0834	-	Glutamine ABC transporter, permease protein
F	Q8CPJ6	COG0044	pyrC	Dihydroorotase
F	Q8CMQ7	COG0517	guaB	Inosine-5’-monophosphate dehydrogenase
H	Q8CNU2	COG0108	ribBA	Riboflavin biosynthesis protein
H	Q8CQV7	COG0214	pdxS	Pyridoxal biosynthesis lyase
H	Q8CNB8	COG1052	pdxB	Putative 2-hydroxyacid dehydrogenase
H	Q8CNZ1	COG0001	hemL	Glutamate-1-semialdehyde 2,1-aminomutase
H	Q8CPQ6	COG1169	-	Isochorismate synthase
P	Q8CR71	COG0855	Ppk	Polyphosphate kinase
P	Q8CPN8	COG1122	-	ABC transporter, ATP-binding protein
P	Q8CPD0	COG0753	catA	Catalase
P	P0C0Q6	COG0605	soda	Superoxide dismutase
P	Q8CPD0	COG0753	katA	Catalase
P	Q8CN76	COG0223	narT	Nitrate ABC transporter substrate-binding protein
R	Q8CNT0	COG1106	-	Abortive phage resistance protein
R	Q8CQ56	COG1064	adhP	Acetaldehyde reductase
R	Q8CMY4	COG0579	mqo4	Probable malate:quinone oxidoreductase 4
R	Q8CTE4	COG0457	-	Uncharacterized protein
R	Q8CPB7	COG1942	-	4-Oxalocrotonate tautomerase
S	Q8CT08	COG4493	-	UPF0637 protein HMPREF9956_0818
-	Q8CMZ9	nog70990	isaA	Putative transglycosylase IsaA
-	Q8CMZ9	NOG70990	gseF	Transglycosylase-like domain protein
-	Q7CCK7	-	-	Phenol soluble modulin beta 1/beta 2
-	Q8CTA5	-	sufD	FeS assembly protein SufD
-	Q8CQK8	NOG27742	-	Poly (glycerol-phosphate) alpha-glucosyltransferase
-	Q8CS14	NOG28792	-	Uncharacterized protein
-	Q8CTN6	-	-	Uncharacterized protein
-	Q8CU14	-	-	Putative uncharacterized protein

**Table 2:** Proteins found in 983EV.

Class	Accession	COG	Gene Name	Function
J	Q8CS54	COG0522	*rpsD*	30S ribosomal protein S4
J	Q8CRG6	COG0092	*rpsC*	30S ribosomal protein S3
J	Q5HQE9	COG1514	*-*	2’,5’ RNA ligase family protein
L	Q8CSH8	COG0776	*-*	Transcriptional regulator
M	Q8CP74	COG0744	*-*	Transglycosylase
U	Q8CPZ2	COG0653	*secA1*	Protein translocase subunit
C	Q8CNJ3	COG0711	*atpF*	ATP synthase subunit b
C	Q8CT13	COG0508	*pdhC*	Dihydrolipoyllysine-residue acetyltransferase component of pyruvate dehydrogenase complex
C	Q8CPN3	COG1071	*pdhA*	Pyruvate dehydrogenase E1 component, alpha subunit
C	Q8CSL9	COG0508	*odhB*	Dihydrolipoyllysine-residue succinyltransferase, E2 component
G	Q8CPY5	COG0057	*gapA1*	Glyceraldehyde-3-phosphate dehydrogenase, type I
E	Q8CSR8	COG0174	*glnA*	Putative uncharacterized protein
F	Q7CCJ0	COG0503	*purR*	Purine operon repressor
F	Q8CRN4	COG0035	*Upp*	Uracil phosphoribosyltransferase
F	Q8CPC9	COG0516	*guaC*	GMP reductase
F	Q5HL04	COG1328	*nrdD*	Anaerobic ribonucleoside-triphosphate reductase
H	Q8CRN9	COG2145	*thiM*	Hydroxyethylthiazole kinase
H	Q8CRM3	COG5146	*coaW*	Type II pantothenate kinase
P	Q5HM52	COG4594	*fecB*	Periplasmic-binding protein
P	Q8CTM7	COG0025	*-*	Putative Na+/H+ antiporter
R	Q8CN54	COG1380	*lrgA*	Antiholin-like protein LrgA
R	Q8CMN2	COG3942	*sle1*	N-acetylmuramoyl-L-alanine amidase
S	Q8CRV3	NOG42366	*ytxH*	Uncharacterized protein
S	Q8CS40	NOG41643	*-*	Uncharacterized protein
S	Q5HMW0	NOG08342	*-*	Uncharacterized protein
S	Q5HM45	NOG40448	*-*	Putative uncharacterized protein
S	Q5HMK5	NOG80633	*-*	Uncharacterized protein
S	Q8CN89	NOG131938	*-*	Putative uncharacterized protein
S	Q5HMK1	NOG249085	*-*	Uncharacterized protein
-	Q5HMJ2	-	*-*	Uncharacterized protein
-	Q5HMJ9	-	*-*	Uncharacterized protein
-	Q5HMK0	-	*-*	Uncharacterized protein
-	Q7CCK7	-	*-*	Antibacterial protein 2
-	J0ZSL7	-	*-*	Siphovirus Gp157
-	Q5HK77	-	*-*	Methylase S
-	Q8CT20	NOG44554	*-*	Putative uncharacterized protein
-	Q8CRB3	-	*-*	Membrane protein
-	Q8CTN6	-	*-*	Uncharacterized protein

**Table 3:** Gene Ontology (GO) biological process.

GO ID	GO Term	ATCC12228EV	983EV
		Number of Molecules	Number of Molecules
GO:0006412	Translation	28	10
GO:0044267	cellular protein metabolic process	31	11
GO:0006091	generation of precursor metabolites	13	6
GO:0019538	protein metabolic process	31	11
GO:0046365	monosaccharide catabolic process	10	4
GO:0006006	glucose metabolic process	11	4
GO:0006096	Glycolysis	8	4
GO:0005996	monosaccharide metabolic process	12	4
GO:0016052	carbohydrate catabolic process	12	4
GO:0006007	glucose catabolic process	9	4
GO:0019318	hexose metabolic process	11	4
GO:0019320	hexose catabolic process	9	4
GO:0034645	cellular macromolecule biosynthetic process	35	12
GO:0008152	metabolic process	102	0
GO:0009056	catabolic process	28	0
GO:0006099	tricarboxylic acid cycle	6	0
GO:0046356	acetyl-CoA catabolic process	6	0
GO:0009059	macromolecule biosynthetic process	35	0
GO:0009109	coenzyme catabolic process	6	0
GO:0009405	Pathogenesis	0	3
GO:0009987	cellular process	0	33
GO:0010467	gene expression	0	12
GO:0051704	multi-organism process	0	3
GO:0044238	primary metabolic process	0	28
GO:0009112	nucleobase metabolic process	0	2

## Data Availability

Not applicable.

## Supplementary Materials

The following are available online at https://www.mdpi.com/article/10.3390/ijms222313029/s1.
