# Supplementary material for: The Extracellular Vesicles from the Commensal Staphylococcus Epidermidis ATCC12228 Strain Regulate Skin Inflammation in the Imiquimod-Induced Psoriasis Murine Model
Source: Int J Mol Sci. 2021 Dec 2;22(23):13029. doi: 10.3390/ijms222313029 (PMC8657977; doi:10.3390/ijms222313029)
Supplement: Supplementary file 1 [file ijms-22-13029-s001.zip › ijms-1457933-supplementary/ijms-1457933-Supplementary figure S1.pdf]

Supplementary figures

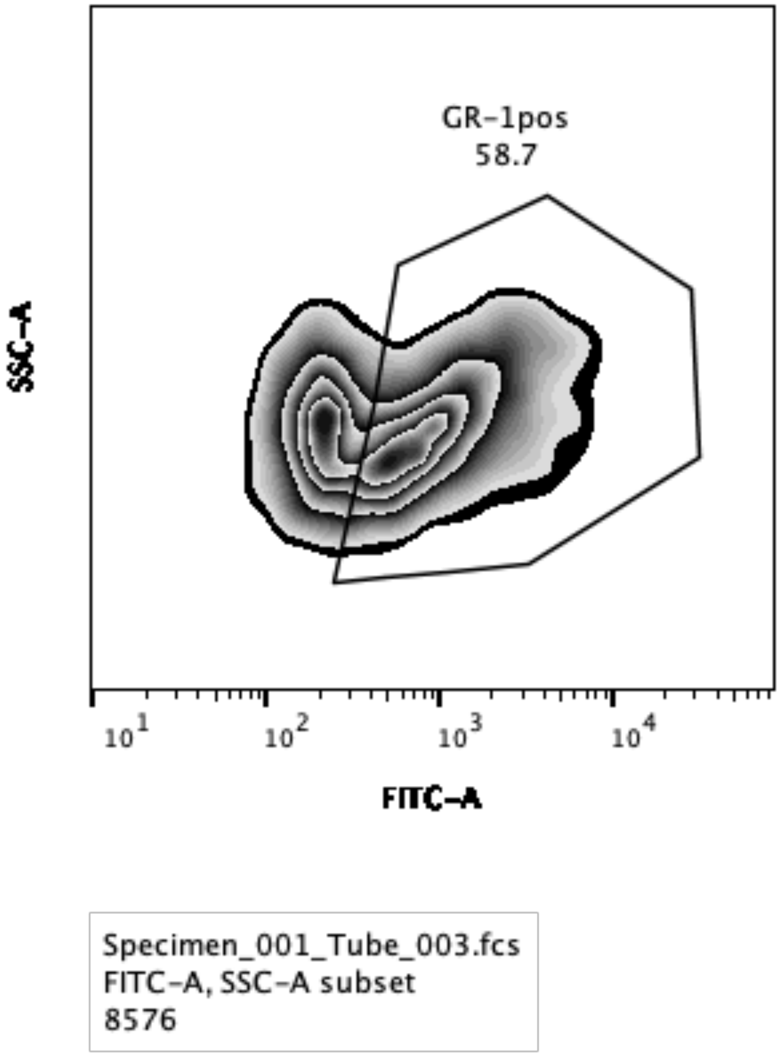

Figure S1. Example of GR1 positive cells analysis from mice skin suspension.
