# Supplementary material for: The Extracellular Vesicles from the Commensal Staphylococcus Epidermidis ATCC12228 Strain Regulate Skin Inflammation in the Imiquimod-Induced Psoriasis Murine Model
Source: Int J Mol Sci. 2021 Dec 2;22(23):13029. doi: 10.3390/ijms222313029 (PMC8657977; doi:10.3390/ijms222313029)
Supplement: Supplementary file 1 [file ijms-22-13029-s001.zip › ijms-1457933-supplementary/ijms-1457933-Supplementary Table S1.pdf]

Table S1. Molecular function

GO ID	GO term	ATCC12228EV		983EV	
		Number/ total	P-value	Number/ total	P-value
GO:0003735	structural constituent of ribosome	24/57	3.0E-14	9/57	2.5E-06
GO:0005198	structural molecule activity	24/58	4.9E-14	9/58	2.9E-06
GO:0019843	rRNA binding	19/40	1.5E-12	7/40	2.0E-05
GO:0003723	RNA binding	23/104	2.9E-07	8/104	0.0018
GO:0000049	tRNA binding	7/18	0.00013	2/18	0.0627
GO:0005488	Binding	77/868	0.00159	0	0
GO:0016903	oxidoreductase activity, acting on the aa.	6/20	0.00202	3/20	0.0098
GO:0003676	nucleic acid binding	36/332	0.0034	11/332	0.1204
GO:0016614	oxidoreductase activity, acting on CH-OH	8/43	0.00952	0	0
GO:0016491	oxidoreductase activity	22/192	0.0139	6/192	0.2756
GO:0016620	oxidoreductase activity, acting on the aa.	4/14	0.01438	1/14	0.2796
GO:0019842	vitamin binding	7/42	0.02688	2/42	0.252
GO:0016616	oxidoreductase activity, acting on the C.	6/35	0.03497	0	0
GO:0048037	cofactor binding	13/114	0.05835	0	0
GO:0030170	pyridoxal phosphate binding	5/33	0.08246	0	0
GO:0070279	vitamin B6 binding	5/33	0.08246	0	0
GO:0000287	magnesium ion binding	6/44	0.09024	2/44	0.2694
GO:0004527	exonuclease activity	3/16	0.1019	0	0
GO:0036094	small molecule binding	36/415	0.10289	0	0
GO:0015078	hydrogen ion transmembrane transporter	0	0	2/16	0.0506
GO:0015077	monovalent inorganic cation transmembran	0	0	2/25	0.1113
GO:0005525	GTP binding	0	0	2/31	0.1585
GO:0019001	guanyl nucleotide binding	0	0	2/31	0.1585
GO:0032561	guanyl ribonucleotide binding	0	0	2/31	0.1585
GO:0016746	transferase activity, transferring acyl	0	0	3/63	0.1745
GO:0022890	inorganic cation transmembrane transport	0	0	2/33	0.175
GO:0008135	translation factor activity, nucleic acid	0	0	1/12	0.2449
