# Supplementary material for: The Extracellular Vesicles from the Commensal Staphylococcus Epidermidis ATCC12228 Strain Regulate Skin Inflammation in the Imiquimod-Induced Psoriasis Murine Model
Source: Int J Mol Sci. 2021 Dec 2;22(23):13029. doi: 10.3390/ijms222313029 (PMC8657977; doi:10.3390/ijms222313029)
Supplement: Supplementary file 1 [file ijms-22-13029-s001.zip › ijms-1457933-supplementary/ijms-1457933-Supplementary Table S2.pdf]

Table S2. Oligonucleotide sequences.

Supplementary Table 2. Oligonucleotides

ID	Sequence
HsGADPH	FW GGTCATCCATGACAACTTTGG
	RV GGAAATGAGCTTGAC
HsVEGFA	FW ATGAACTTTCTGCTGTCTTCGGT
	RV TGGCCTTGGTGAGGTTTGATCC
HsLL-37	FW ATGAAGACCCAAAGGGATGGCC
	RV CTAGGACTCTGTCCTGGGTACAAG
HsIL-6	FW CAGCCACTCACCTCTTCAGAACG A
	RV CTGGCATTTGTGGTTGGGTCAGG
HsIL-8	FW AGTTTTTGAAGAGGGCTGAGA
	RV ACCAAGGCACAGTGGAACAA
HsIL-10	FW ATGCCCCAAGCTGAGAACCAAGACCTA
	RV TCTCAAGGGGCTGGGTCAGCTATCCCA
HsIL-17F	FW ATGCCCCAAGCTGAGAACCAAGACCTA
	RV TCTCAAGGGGCTGGGTCAGCTATCCCA
MmGADPH	FW CTACCCCCAATGTGTCCGTC
	RV GCCGTATTCATTGTCATACCAGG
MmVEGFA	FW CTTGCAGATGTGACAAGCCAA
	RV AGCAGCAGATATAAGAAAATGGCG
MmIL-6	FW ACCAGAGGAAATTTTCAATAGGC
	RV TGATGCACTTGCAGAAAACA
MmMIP2	FW CCCAGACAGAAGTCATAGCCAC
	RV CTTCCGTTGAGGGACAGCAG
MmKC	FW CCCAAACCGAAGTCATAGCCA
	RV CTCCGTTACTTGGGGACACC
MmIL-17F	FW CAAGAAATCCTGGTCCTTCG
	RV GAGCATCTTCTCCAACCTGAA
MmFOXP3	FW GAACCCAATGCCCAACCCTA
	RV ATCTGCTTGGCAGTGCTTGA
MmIL-36α	FW GCAAACAGTTCCAGTCACTAT
	RV GGGTGTCTTTGATTGCTTCTT
MmIL-36β	FW TGCATGGATCCTCACAATC
	RV TGCATGGATCCTCACAATC
MmIL-36ɣ	FW CACAGAGTAACCCCAGTCAG
	RV TTGGTCCTGCTTACCTTTCA
MmIL-36R	FW GTCCTTCAGACCTCTCCTG
	RV CGGTTAGGTTCACAGCTATTT
MmIL-36RA	FW TCCAGCCTTGTCTTTGCCTAAA
	RV CCCACAAGATAGGCACTGGC
Hs, Homo Sappiens; Mm, Mus musculus.	
